# Comparison of In Vitro Biotransformation of Olive Polyphenols Between Healthy Young and Elderly

**DOI:** 10.3390/metabo15010026

**Published:** 2025-01-07

**Authors:** Stef Lauwers, Anne-Sophie Weyns, Annelies Breynaert, Tim Van Rillaer, Valerie Van Huynegem, Erik Fransen, Wout Bittremieux, Sarah Lebeer, Emmy Tuenter, Nina Hermans

**Affiliations:** 1 Natural Products & Food Research and Analysis—Pharmaceutical Technology (NatuRAPT), University of Antwerp, 2610 Wilrijk, Belgium; 2 Laboratory of Applied Microbiology and Biotechnology, Department of Bioscience Engineering, University of Antwerp, 2020 Antwerp, Belgium; 3 Center for Medical Genetics, Faculty of Pharmaceutical, Biomedical and Veterinary Sciences, University of Antwerp, 2650 Edegem, Belgium; 4 ADReM Data Lab, Department of Computer Science, University of Antwerp, 2020 Antwerp, Belgium

**Keywords:** oleuropein, olive polyphenols, gastrointestinal biotransformation, metabolomics, gut microbiome

## Abstract

Background: Olive leaves are a rich source of polyphenols, predominantly secoiridoids, flavonoids, and simple phenols, which exhibit various biological properties. Extracts prepared from olive leaves are associated with hypoglycemic, hypotensive, diuretic, and antiseptic properties. Upon ingestion, a substantial fraction of these polyphenols reaches the colon where they undergo extensive metabolism by the gut microbiota. Host characteristics, like age, can influence the composition of the gut microbiome, potentially affecting the biotransformation of these compounds. Therefore, it can be hypothesised that differences in the gut microbiome between young and elderly individuals may impact the biotransformation rate and the type and amount of metabolites formed. Methods: An in vitro biotransformation model was used to mimic the conditions in the stomach, small intestine and colon of two age groups of healthy participants (20–30 years old, ≥65 years old), using oleuropein as a single compound and an olive leaf extract as test compounds. The bacterial composition and metabolite content were investigated. Results: The study revealed that, while the same metabolites were formed in both age groups, in the young age group, less metabolite formation was observed, likely due to a reduced viable cell count. Most biotransformation reactions took place within the first 24 h of colon incubation, and mainly, deglycosylation, hydrolysis, flavonoid ring cleavage, and demethylation reactions were observed. A bacterial composition analysis showed a steep drop in α-diversity after 24 h of colon incubation, likely due to favourable experimental conditions for certain bacterial species. Conclusions: Both age groups produced the same metabolites, suggesting that the potential for polyphenols to exert their health-promoting benefits persists in healthy older individuals.

## 1. Introduction

Throughout history, the olive tree (*Olea europaea* L., Oleaceae) played a prominent part in society, especially in the Mediterranean region. Its dominant spiritual and symbolic role is reflected by its frequent mention in myths and religious scriptures [1]. The first signs of cultivation of the olive tree date back to the Early Bronze Age (ca. 3000 BC) [2]. The tree was, and still is, mainly grown for its fruits, i.e., olives, that were harvested and turned into olive oil or table olives [1]. Aside from its culinary use, almost every part of the olive tree has been used in traditional medicine. The bark, fruits, leaves, wood, seeds, and oil are used in different preparations and are claimed to help in a wide variety of illnesses. Olive oil was used to treat small wounds and burns, help with gallstones, or work as a laxative. Infusions of the olive leaves are claimed to have hypoglycemic, hypotensive, diuretic, and antiseptic properties [3,4,5]. This plethora of traditional uses makes olive leaves and fruits fascinating research subjects.

Olive products and their constituents have been extensively studied, mostly for their polyphenol content. Polyphenols are secondary plant metabolites that have been associated with a large variety of health-promoting effects, including antioxidant, anti-inflammatory, anticarcinogenic, and antiseptic activities [6]. Notwithstanding the beneficial effects of polyphenols, genotoxicity (in vitro and in vivo) and liver and thyroid toxicity (in vivo) have been reported. It is important to mention that polyphenol consumption via food products is not expected to result in levels high enough to exert such toxicity effects, but some manufacturers of polyphenol supplements recommend an intake that greatly exceeds the polyphenol intake associated with the diet [7]. Olive leaves are a rich source of polyphenols and, therefore, possess the highest antioxidant and scavenging power among the different parts of the olive tree. Compounds, such as secoiridoids (oleuropein and verbascoside), flavonoids (luteolin-7-*O*-glucoside, apigenin-7-*O*-glucoside, and rutin), and simple phenols, like hydroxytyrosol and vanillic acid, have been detected in olive leaves [8,9]. The ester of elenolic acid glucoside and hydroxytyrosol, oleuropein, is the most abundant in olive leaves, where its concentration ranges from 1 to 14% (*w*/*w*) [9]. Hydroxytyrosol is the main degradation product of oleuropein. The conversion of oleuropein into hydroxytyrosol happens through chemical and enzymatic reactions during fruit ripening and olive oil production, making hydroxytyrosol the most abundant polyphenol in olive fruits and olive oil [10].

Most polyphenols show low bioavailability, leading to discrepancies in explaining their many beneficial effects [11]. Because of the poor absorption, a large portion will pass through the large intestine, where they will be extensively metabolised by the colonic microbiome, supporting the hypothesis that the biological effects may be mediated by their metabolites [11,12]. The colon accommodates 1000–1250 kinds of bacterial species, reaching concentrations of up to 10^10^–10^12^ colony-forming units (CFU) per gram, which can interact with humans in various ways, such as the immune system and the biotransformation of food and xenobiotics [13,14,15]. Gut microbes, such as *Escherichia*, *Bifidobacterium*, *Eubacterium*, *Lactobacillus*, *Lacticasibacillus*, *Lactiplantibacillus*, *Leimosilactobacillus*, *Bacteroides*, and *Streptococcus,* participate in the biotransformation of natural products. These bacteria can metabolise natural products, like polyphenols, through hydrolysis, methylation, demethylation, redox, and cyclisation reactions, producing different metabolites [15,16]. The literature previously described the deglycosylation of oleuropein and flavonoid–glycosides. Sequentially, the aglycones are further metabolised into smaller phenolics through hydrolysis or ring cleavage reactions. The resulting small phenolics can undergo additional biotransformation reactions, such as reduction, methylation or demethylation reactions [15,17,18].

This gut microbiome can be influenced by external factors like diet and lifestyle, as well as by host characteristics like host health, sex, genetics, and age [19,20]. The increased life expectancy and the ageing population in the Western world make investigating the changes in the gut microbiome by age increasingly relevant. This is especially true when polyphenols can have beneficial effects in age-related diseases, e.g., neurodegenerative, metabolic, and cardiovascular diseases.

Finally, the dissimilarity in colon microbial composition may have the most substantial impact on the difference in polyphenol biotransformation between healthy young and healthy elderly individuals. Therefore, it can be hypothesised that differences can be observed between the biotransformation rate of polyphenols and the type and amount of metabolites formed among these two population groups.

This paper aims to compare the biotransformation of olive leaf polyphenols between these two age groups, which is an important determinant in the eventual pharmacological effects observed for these olive polyphenols.

## 2. Materials and Methods

### 2.1. Chemicals

Ultra-high-performance liquid chromatography (UHPLC)-grade MeOH, acetonitrile, and formic acid were purchased from Biosolve (Dieuze, France). The following analytical standards were obtained from Sigma-Aldrich (St. Louis, MO, USA): apigenin, benzoic acid, caffeic acid, catechin, chlorogenic acid, cinnamic acid, coumarin, 3,4-dihydroxyphenylacetic acid, 3,4-dihydroxyphenylpropionic acid, epicatechin, ferulic acid, homovanillic acid, hydroxyhippuric acid, 4-hydroxyphenylacetic acid, 3-(4-hydroxyphenyl)propionic acid, hydroxytyrosol, isorhamnetin, naringenin, *p*-coumaric acid, protocatechuic acid, quercetin, quercitrin, quinic acid, rutin, salicylic acid, sinapic acid, syringic acid, tannic acid, theophylline, tyrosol, and vanillic acid. Luteolin and procyanidin B2 were provided by Santa Cruz Biotechnology (Santa Cruz, CA, USA). Apigenin-7-*O*-glucoside, gallic acid, luteolin-7-*O*-glycoside, oleuropein, and *p*-hydroxybenzoic acid were provided by Carl Roth (Karlsruhe, Germany). The olive leaf extract was a hydroethanolic extract provided by Tilman (Baillonville, Belgium). Pepsin from porcine gastric mucosa, pancreatin from porcine pancreas, porcine bile extract, glycerol, and thioglycolate broth were purchased from Sigma-Aldrich (St. Louis, MO, USA). Hydrochloric acid (32%), NaHCO_3_, and Na_2_HPO_4_ were purchased from Fisher Scientific (Loughborough, UK). NaH_2_PO_4_.2H_2_O and NaOH were purchased from Merck (Darmstadt, Germany). Milli-Q water was generated using the Direct-Pure Water UP System from RephiLe Bioscience (Boston, MA, USA).

### 2.2. Extract Identification

#### 2.2.1. UPLC-QTOF-MS Analysis

Characterisation of the olive leaf extract was performed through analysis with an ACQUITY LC system coupled to a Xevo G2-XS QTof spectrometer (Waters, Milford, MA, USA). The extract was dissolved and diluted to a final concentration of 100 µg/mL in MeOH–water (10:90 *v*/*v*). An aliquot of 5 µL was injected into a Waters Acquity UPLC BEH C18 (2.1 × 100 mm, 1.8 µm) column, which was kept at 40 °C. The mobile phase solvents consisted of water + 0.001% formic acid (A) and acetonitrile + 0.001% formic acid (B). The gradient was set as follows: 0 min, 3% B; 3 min, 7% B; 6 min, 15% B; 9 min 25% B; 12 min, 50% B; 14 min, 70% B; 15 min, 100% B; 17 min, 100% B; 19 min, 3% B; and 22 min, 3% B. The flow rate was set at 0.4 mL/min. Detection and accurate mass measurements were conducted in ESI(−) MS^E^ mode, with recorded *m*/*z* values from 50 to 1500. The capillary voltage was set to −0.8 kV and the cone voltage to 10 V; the cone gas flow and desolvation gas flow at 50 L/h and 1000 L/h, respectively; and the source temperature and desolvation temperature at 120 °C and 500 °C, respectively. A ramp collision energy from 20 V to 30 V was applied to obtain additional structural information. Leucine encephaline was used as a lock mass.

Stock solutions of analytical standards were prepared at a concentration of 1 mg/mL in UPLC-grade MeOH and stored at −80 °C. A mixture of the analytical standards was prepared from the stock solutions and diluted to a final concentration of 0.625 µg/mL with MeOH:H_2_O (10:90 *v*/*v*).

#### 2.2.2. Data Analysis

The data were processed with MassLynx software, version 4.1. The compounds were identified based on their *m*/*z* values, retention time, and fragmentation patterns and comparing them to analytical standards and literature data. Identified compounds were assigned a level of confidence as proposed by Schymanski et al. [21].

### 2.3. Gastrointestinal Biotransformation

An in vitro gastrointestinal model was used to simulate human biotransformation processes in the stomach, small intestine, and colon. The digestive juices and faecal suspension were composed to mimic the human conditions of two age groups: healthy young individuals between 20 to 30 years old and healthy older individuals of 65 years or older. This model was developed and validated in-house, as previously reported [22,23,24].

#### 2.3.1. Collection and Processing of the Faecal Samples

To investigate the difference in biotransformation between young and elderly individuals, faecal samples of twelve healthy donors from the two age groups were collected. Each group consisted of six male and six female donors that met the following inclusion criteria: age between 20 and 30 years old or at least 65 years old (for the young and elderly age groups, respectively), body mass index (BMI) between 18.5 and 25, waist circumference <88 cm for women and <102 cm for men, not pregnant or menstruating, non-smoking, non-vegetarian or non-vegan, normal defecation, no history of gastrointestinal disease, and no intake of anti-, pre-, or probiotics three months prior to donation. A complete list of the inclusion criteria can be found in the Supplementary Materials, Section S1. Ethical approval for the collection of the faecal samples was obtained from the Ethical Committee of the Antwerp University Hospital (reference number: 20/35/444).

The preparation of the faecal suspension was executed according to a previously developed protocol [23]. In short, the donors collected faecal samples using Protocult collection containers (Ability Building Center, Rochester, MN, USA), which were kept at room temperature with an anaerocult bag from Merck (Darmstadt, Germany). Within 4 h after collection, a 10% (*w*/*v*) faecal slurry was prepared in a sterile phosphate buffer (0.1 M, pH 7.0, 0.58% *w*/*v* Na_2_HPO_4_, 1.03% *w*/*v* NaH_2_PO_4_.2H_2_O, and 3.45% *w*/*v* thioglycolate broth) combined with glycerol (17% *v*/*v*) in an anaerobic glove box (Jacomex Glove Box T3, TCPS, Rotselaar, Belgium). The homogenisation and elimination of solid particles were performed by a Stomacher^®^ lab blender (VWR, Leuven, Belgium) for three minutes. Aliquots of 20 mL of faecal suspension were stored at −80 °C until further use. All samples were registered and stored in the Biobank Antwerp, Antwerp, Belgium [25].

#### 2.3.2. Simulation of Stomach, Small Intestine, and Colon

In this experiment, three sample types were included: (1) samples containing the extract or compound of interest, in this case 200 mg olive leaf extract (OLIVEL) or 25 mg of oleuropein (OLE), prepared in triplicate; (2) negative control samples, also containing the extract or compound of interest and digestive enzymes, but no faecal matter (NCOLIVEL or NCOLE), prepared in duplicate; and (3) method blanks not containing any extract or compound (BL), prepared in duplicate.

Gastrointestinal biotransformation was simulated with a previously *in-house* developed and validated gastro-intestinal biotransformation model with colon phase [22,24]. During the experiment, human biotransformation processes in the stomach, small intestine, and colon were mimicked by adjusting the pH levels and adding the corresponding enzymes and pooled human faecal suspensions. During the colon phase, the samples were incubated for 72 h at 37 °C.

For UPLC-ESI-QToF analysis, the samples were taken at the start of the experiment (T0), after the stomach phase (G), after the small intestinal phase (SI), and after 2 h, 4 h, and 6 h and then every 6 h of colon incubation (C2–C72).

The experiment was executed with conditions mimicking the gastrointestinal digestion of young individuals, as described above, and resembling conditions in elderly individuals. For the latter age group, modifications to the protocol were made according to the literature [26]. The pepsin concentration was reduced by 35%, resulting in a pepsin solution of 404.300 FIP-U/100 mL, 0.1 M HCl, and a faecal pool of the corresponding age group was added to the samples during the colon phase.

To assess the validity of the experiment, chlorogenic acid was used as a positive control (PC) [22]. An explanation of the use of the PC samples can be found in the Supplementary Materials, Section S2.

Viable cell concentrations were monitored during colon incubation. Samples of the start (C0) and after 72 h of colon phase (C72) were diluted using a decimal dilution series and plated onto TSA (Tryptic Soy Agar, Sigma-Aldrich, St. Louis, MO, USA) plates. The plates were prepared by suspending 40 g of TSA powder in 1 L H_2_O. After sterilisation, the plates were poured with a thickness of 0.5 cm. A decimal dilution series of each sample was prepared, ranging from 10^−1^–10^−12^ CFU/mL with DPBS (Dulbecco’s Phosphate Buffered Saline, Fisher Scientific, Hampton, NH, USA). A volume of 15 µL of the 10^−3^ to 10^−12^ CFU/mL dilutions was plated out in triplicate and incubated under anaerobic conditions at 37 °C for 48 h. The CFUs were counted after 24 h and 48 h. A statistical analysis was conducted to investigate the differences in viable cell count was performed with SPSS (IBM, version 29.0.1.0).

#### 2.3.3. UPLC-ESI-QTOF Analysis

Samples from the biotransformation experiments were analysed by means of UPLC-ESI-QToF mass spectrometry in negative ion mode. The applied method is described in paragraph 2.2.1. To add to this protocol, a dilution series of the standard solution, with concentrations ranging from 39 ng/mL to 1.25 µg/mL, was injected at the start and the end of the run. To monitor analytical drift and assess precision, quality control (QC) samples were injected after every two time points. All dilutions were made with MeOH:H_2_O (10:90 *v*/*v*) as the diluent.

The PC samples were analysed with the same column but with a different method, as previously described [24].

#### 2.3.4. Data Analysis

The automated data analysis workflow used was developed and validated by Beirnaert et al. [27] and Peeters et al. [28]. Briefly, by using the XCMS and EDGE packages in R and a random forest machine-learning model called tinderesting, time profiles of *m*/*z* values with significant differences between blank (BL), negative control (NC), and test samples (OLE/OLIVEL) were created and scored from 0 to 1. Features with a tinderesting score of 0.8 or higher were manually checked and annotated by using their exact *m*/*z* values, retention time, and fragmentation patterns and comparing them to analytical standards, the literature, and spectral databases whenever feasible. Chromatograms were processed with MassLynx software, version 4.1. Software for in silico metabolite prediction BioTransformer 3.0 [29,30] was used to generate possible human gut microbial biotransformation products for a suspect screening method. The SMILES string of oleuropein and other compounds, identified in the extract with a confidence level 1 or 2, were uploaded and “Human Gut Microbial Transformation” with 2 reaction iterations was selected.

In the PC samples, the aforementioned metabolites were identified using analytical standards. Time profiles of chlorogenic acid and its metabolites were produced to confirm the in vitro biotransformation.

Marvin, version 23.1.87 (ChemAxon, Budapest, Hungary) [31] was used for drawing, displaying, and characterising the chemical structures, substructures, and reactions.

### 2.4. Sequencing

To assess the bacterial composition of the individual faecal samples before pooling, 16S rRNA V1-V9 gene sequencing was performed. At the start and after 24 h, 30 h, 48 h, and 72 h of colon incubation, samples were taken of the method blanks (BL), olive leaf extract (OLIVEL), and oleuropein (OLE) samples of the biotransformation experiment. These samples were sequenced through 16S rRNA Illumina sequencing of hypervariable region V4. The bacterial composition and alpha (α) and beta (β) diversity were assessed.

#### 2.4.1. Microbial DNA Isolation

Microbial DNA was extracted from the faecal suspensions of the individual faecal samples using the FastDNA™ SPIN Kit (MP Biomedicals, Irvine, CA, USA) according to the manufacturer’s instructions. The concentration of DNA isolates was quantified by a Qubit 2.0 Fluorometer with the dsDNA HS Assay kit (ThermoScientific, Waltham, MA, USA).

#### 2.4.2. 16S rRNA Gene Amplification and Sequencing

Quantitative PCR (qPCR) was used for the estimation of absolute bacterial, fungal, and human DNA concentrations in samples after extraction. Illumina MiSeq 16S rRNA gene amplicon sequencing was performed as described by Weyns et al. [32]. The obtained merged and denoised reads (amplicon sequence variants or ASVs) were taxonomically annotated from kingdom to species level using a 16S rRNA reference database constructed from a combination of the GTDB release 214 Small Sub-Unit (SSU) gene reference data [33,34] and the 16S sequences of Eukaryota in the Silva release 138.2 SSU [34]. All data handling and visualisation were performed in R using the tidyverse set of packages and tidyamplicons package (https://github.com/Swittouck/tidyamplicons (accessed on 15 February 2024)).

### 2.5. Comparison Between Age Groups

#### 2.5.1. Comparison of Metabolite Profiles

To compare the biotransformation patterns of the metabolites between the two age groups, peak areas of identified compounds with confidence levels 1, 2, or 3 of each time point were determined using TargetLynx software, version 4.1.

To assess the difference in overall concentration across all time points between the two age groups, the area under the curve (AUC) for each replicate of each metabolite was calculated using the trapezoid rule, as implemented in the R package pracma, version 2.4.4 [35]. The applied statistical methods are described in Section 2.6.

#### 2.5.2. Comparison of Bacterial Composition

After 0 h, 24 h, 30 h, 48 h, and 72 h of colon incubation, samples were taken from the BL, OLE, and OLIVEL samples. A sequencing analysis was performed with the method described in Section 2.4. Quality control of the data was performed by determining the amount of non-bacterial reads and evaluating the read length and the amount of reads in the samples.

Afterwards, the relative abundance of the most abundant genera was visualised in stacked bar plots at the genus taxonomical level for the different samples taken during the colon phase to examine the microbial composition. Additionally, the α-diversity, β-diversity, and potential differences in the abundance of specific taxa were investigated. The applied statistical methods are described in Section 2.6.

### 2.6. Statistical Methods

To study the difference in metabolite formation at each separate time point between the two age groups, a one-way ANOVA was carried out on the peak areas of the three replicates at each time point for each of the investigated metabolites. The resulting *p*-values were corrected for multiple testing using the false discovery rate (FDR) method, as implemented in the R package qvalue, version 2.26.0 [36].

To compare the difference in overall concentration across all time points between the two age groups for a certain metabolite, a one-way ANOVA was carried out to investigate the difference in mean AUC between the groups. All statistical tests were carried out in the software package R, version 4.3.2 [37].

When comparing the bacterial composition of the colon samples, the inverse-Simpson α-diversity of the samples between the two age groups was assessed, the β-diversity was investigated through PCoA plots of the Bray–Curtis dissimilarity of the individual samples, and a codifferential abundance analysis was performed to find differences in the abundance of specific taxa between the two age groups. The data handling and visualisation were performed in R using the tidyamplicons package [38].

## 3. Results

### 3.1. Extract Identification

With the described method, a total of 16 phenolic compounds were identified tentatively or with a reference standard. Mainly flavonoids, phenolic acids, secoiridoids, and other olive-specific polyphenols were characterised. In the Supplementary Materials, a detailed description of the identification of the compounds can be found in the corresponding Section S3, and a summary of the identified compounds, showing the proposed name, molecular formula, experimental and calculated *m*/*z* of the [M-H]^−^ adduct, error, fragments, confidence level of identification, and used references, can be found in Supplementary Table S1.

### 3.2. Gastrointestinal Biotransformation

#### 3.2.1. Construction of Faecal Suspension Pool

**Figure 1:**
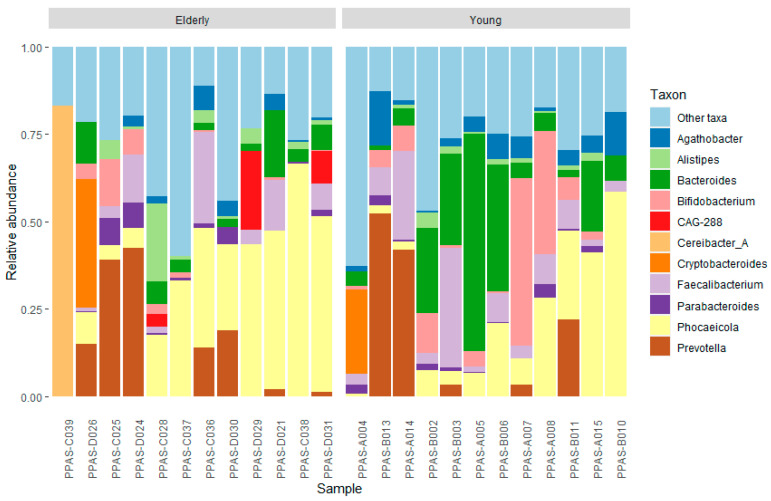
Relative abundance of taxa at genus level in individual faecal samples.

Before pooling, the bacterial composition of the individual faecal samples was determined as described in Section 2.4. A stacked barplot of the relative abundance of the different taxa at the genus level for each sample is depicted in Figure 1. The eleven most abundant genera are displayed separately; the remaining genera are depicted as ‘other’. The purpose of the sequencing of the individual samples is to determine if there are samples with a largely divergent bacterial composition. If this is the case, the sample will not be added to the faecal suspension pool. Overall, *Prevotella* and *Phocaeicola* are the most abundant in samples of both young and elderly populations. *Bifidobacterium* and *Bacteroides* are more present in the samples of young individuals. Sample PPAS-C039 is largely dominated by the genus *Cereibacter*, which makes it substantially different from the other samples and, therefore, was not added to the faecal suspension pool of the elderly population. All samples of the younger individuals were used to prepare the faecal suspension pool of the young population.

#### 3.2.2. Metabolite Identification

Samples of the in vitro gastrointestinal biotransformation of oleuropein and olive leaf extract were analysed with UPLC-ESI-QTOF mass spectrometry to observe the formation and breakdown of metabolites. The identification of the detected biotransformation products of oleuropein, olive-specific polyphenols, flavonoids, and phenolic acids is described below. The same metabolites were identified in the samples of both age groups.

##### Biotransformation of Oleuropein

**Figure 2:**
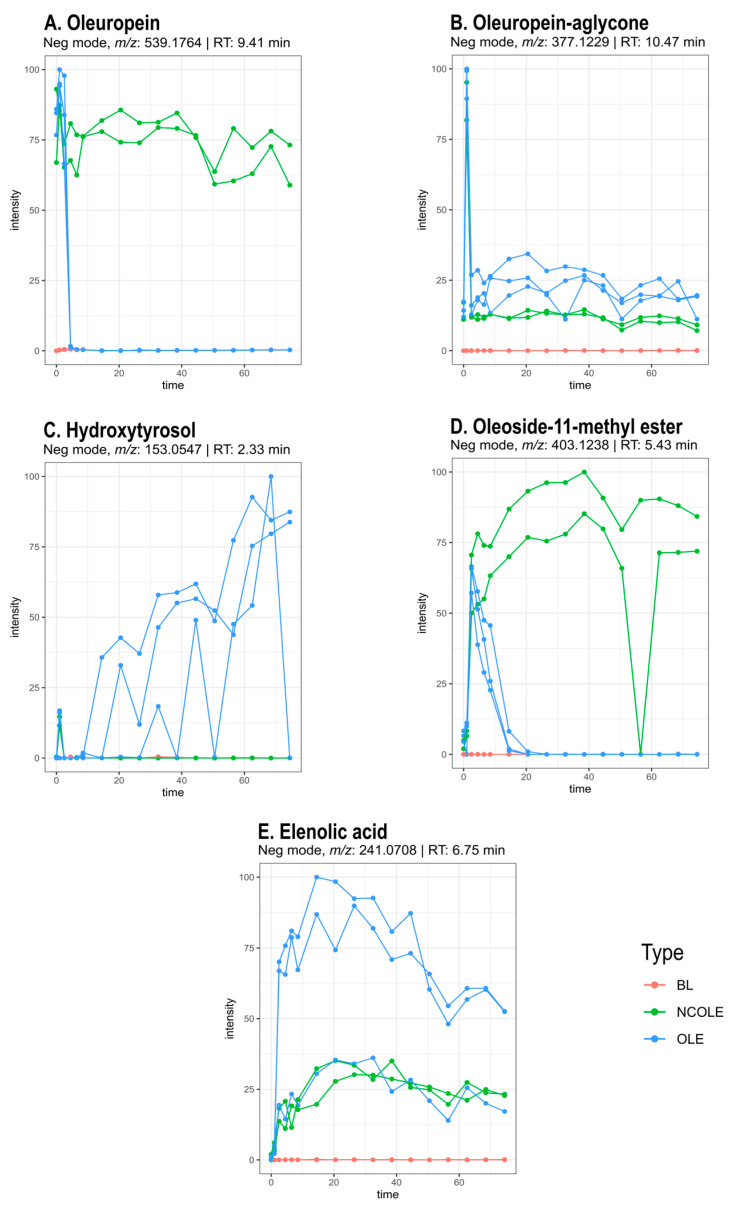
Selection of time profiles of oleuropein (**A**), oleuropein aglycone (**B**), hydroxytyrosol (**C**), oleoside-11-methyl ester (**D**), and elenolic acid (**E**). Time on x-axis is expressed in hours and relative intensity of the detection signal is plotted on the y-axis. Test samples with oleuropein (OLE), negative control samples (NCOLE), and blank samples (BL) are depicted in blue, green, and red, respectively. The metabolites were detected in samples of both age groups.

Oleuropein, as a single compound, was studied during in vitro biotransformation, and its breakdown and the formation of its metabolites were investigated. Oleuropein is the most abundant polyphenol present in olive leaves and consists of hydroxytyrosol, elenolic acid, and glucose. The compound was identified with a reference standard (*m*/*z* 539.1764 [M-H]^−^). The identified metabolites are summarised in Table 1. Upon inspection of the time profile of oleuropein (Figure 2A), a decrease in intensity can be observed over time. A rapid decrease during the small intestinal simulation and the first hours of colon incubation was observed. After 6 h of colon incubation, the signal could not be detected anymore. The signal in the negative control samples (NCOLE), which did not contain any faecal bacteria, persisted during the experiment. In the blank samples (BL), the signal is absent.

By investigating the time profiles of the detected ions that were not present at the start of the experiment but were formed over time, possible metabolites of oleuropein could be identified. Oleuropein aglycone was tentatively annotated to *m*/*z* 377.1229 ([M-H]^−^) by the presence of fragments *m*/*z* 241 and 195 described in the literature [39] and the spectral database HMDB [40]. The time profile (Figure 2B) of this signal in the test samples (OLE) is similar to that of the NC samples. The signal is not present in the blank samples (BL). Formation occurred during the gastric phase, and the compound was degraded during the small intestinal phase and after 2 h of colon incubation. Hydroxytyrosol was identified with a reference standard (*m*/*z* 153.0547 [M-H]^−^). The time profile (Figure 2C) displays an increasing signal of the test samples after 6 to 12 h of colon incubation, while the compound is not detected in the blank and negative control samples. Oleoside-11-methyl ester (*m*/*z* 403.1238 [M-H]^−^) was tentatively identified based on fragments *m*/*z* 223 and 179 described in the literature [39] and HMDB [40]. The time profile of the test samples (Figure 2D) shows an intermediate course, meaning the signal increases over time, reaches a peak, and decreases again until the signal disappears. The highest intensity can be observed after the small intestine phase. The signal is absent after 12 to 18 h of colon incubation. However, in the negative control samples, the signal kept increasing and levelled off after 30 to 36 h of colon incubation. Another increasing profile was observed for the ion with *m*/*z* 241.0708 ([M-H]^−^) (Figure 2E). Based on the predicted molecular formula C_11_H_13_O_6_ ([M-H]^−^), the double-bond equivalent (DBEq) 5.5, mass error 1.66 ppm, and experimental data, this ion was assigned to elenolic acid with confidence level 3.

**Figure 3:**
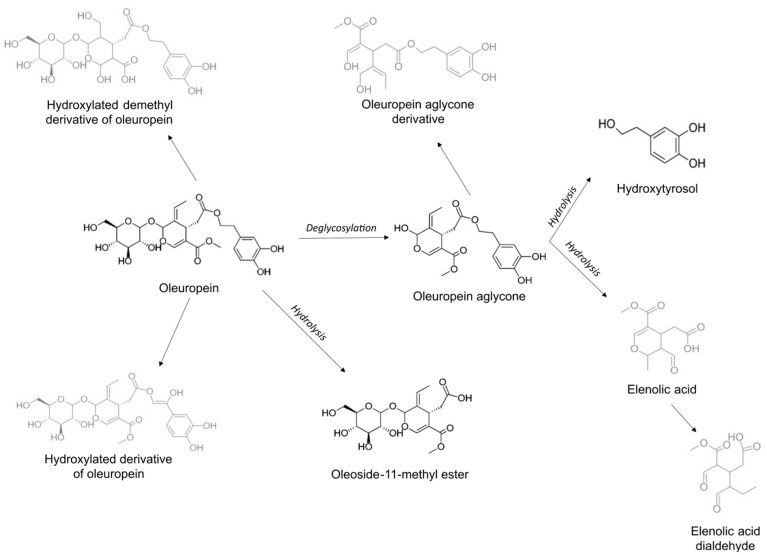
Proposed biotransformation pathway of oleuropein. Structures of compounds identified with confidence levels I and II are depicted in black, and structures of compounds with confidence level III are depicted in grey. The metabolites were detected in samples of both age groups.

Four other compounds were tentatively identified. A hydroxylated derivative of oleuropein *m*/*z* 553.1557 ([M-H]^−^), with fragments *m*/*z* 403 and 223, corresponding with oleoside-11-methyl ester and the elenolic acid moiety, respectively. These fragments are equal to the fragments found in the MS^2^ spectrum of oleuropein, indicating a structural relation. Since the oleoside-11-methyl ester and the elenolic acid moiety are the same as in oleuropein, the structural alteration is most probably situated in the hydroxytyrosol moiety. The difference between the molecular ions of oleuropein (*m*/*z* 539 [M-H]^−^) and its hydroxylated derivative (*m*/*z* 553 [M-H]^−^) equals 14 Da. This can correspond with the addition of a hydroxyl group (16 Da) and a double bond, resulting in the loss of two hydrogen atoms (2 Da). The prediction of the elemental composition yields C_25_H_29_O_14_ as the molecular formula of the [M-H]^−^ molecular ion. The double-bond equivalent (DBEq) of 11.5 and a mass error of 0.0 support this hypothesis. Potential structures are depicted in Figure 3.

The ion with *m*/*z* 547.1650 ([M-H]^−^) is tentatively identified as a hydroxylated demethyl derivative of oleuropein. The mass difference with oleuropein of 8 Da can correspond to the saturation of two carbon–carbon double bonds (+4 Da), the addition of a hydroxyl group (+16 Da), the substitution of the methyl group with a hydroxyl group (+2 Da), and the loss of a methyl group (−14 Da). This proposition is confirmed by the predicted molecular formula C_23_H_31_O_15_ of the molecular [M-H]^−^ ion, its DBEq of 8.5, and the mass error of 2.38. A potential structure is displayed in Figure 3.

A derivative of oleuropein aglycone (*m*/*z* 379.1383 [M-H]^−^) was detected with the molecular formula C_19_H_23_O_8_ of the [M-H]^−^ ion. The potential structure (Figure 3) and the predicted molecular formula are supported by the DBEq (8.5) and the mass error (2.64). In the same manner, the ion with *m*/*z* 243.0862 could be identified as elenolic acid dialdehyde with molecular formula C_11_H_15_O_6_ ([M-H]^−^), DBEq (4.5), and mass error (2.88). A proposed biotransformation pathway of oleuropein is depicted in Figure 3.

##### Biotransformation of Olive-Specific Polyphenols

The olive-specific polyphenols ligstroside (*m*/*z* 523.182 [M-H]^−^), (iso)verbascoside (*m*/*z* 623.1976 [M-H]^−^), and oleuropein–glucoside (*m*/*z* 701.2283 [M-H]^−^) degraded during the colon phase. The time profiles followed the same course as oleuropein. Thus, the compounds were not detected after 6 to 12 h of colon incubation. The signal persisted in the negative control samples. A lactone ester with hydroxytyrosol could be identified with *m*/*z* 321.1332 ([M-H]^−^) and the predicted molecular formula C_17_H_22_O_6_ ([M-H]^−^). The reported fragment *m*/*z* 185 was detected and confirmed by the literature [39,41].

For 13 other ions, a molecular formula and a name were assigned using the MassLynx elemental composition prediction software and the literature [41]. A summary of these and the above-mentioned compounds can be found in Table 2.

##### Biotransformation of Flavonoids

**Figure 4:**
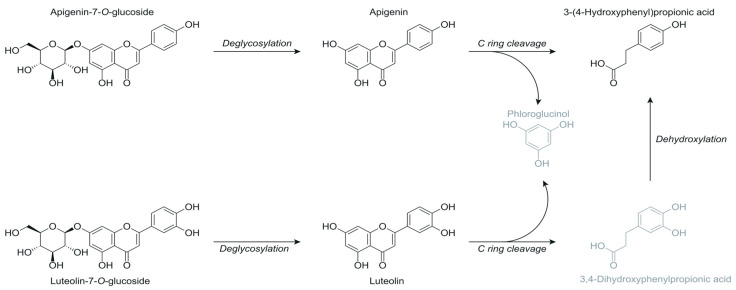
Proposed biotransformation pathway of apigenin-7-*O*-glucoside and luteolin-7-*O*-glucoside. Structures depicted in grey were not detected in the samples. The metabolites were detected in samples of both age groups.

Different flavonoids, such as flavones, flavanones, and flavanols, were detected at the start of the experiment. Mainly the glycosides are present in the beginning, but they are quickly metabolised into their aglycons after 2 to 4 h of colon incubation. This is the case for the flavones luteolin, apigenin, and chrysoeriol. The presence of their glucosides was confirmed with a reference standard or with the literature [39,42] and spectral databases (MassBank.eu) [43,44]. Luteolin (*m*/*z* 285.0394 [M-H]^−^) and apigenin aglycone (*m*/*z* 269.0449 [M-H]^−^) were also identified with a reference standard. Their time profiles showed an intermediate course. Apigenin-7-*O*-rutinoside (*m*/*z* 577.1559 [M-H]^−^) was identified using information from HMDB [40]. Luteolin-7,4-*O*-diglucoside (*m*/*z* 609.1456 [M-H]^−^) was also tentatively identified using the presence of the fragment *m*/*z* 447, corresponding to luteolin–glucoside, from the spectral database MassBank.eu as conformation. Two luteolin–hexosyl–rhamnosides (*m*/*z* 593.1493 [M-H]^−^, rt 7.79, 8.28 min) with the molecular formula C_27_H_29_O_15_ ([M-H]^−^) were identified with fragments *m*/*z* 447 and 285 [39]. Figure 4 proposes the biotransformation pathway of apigenin-7-*O*-glucoside and luteolin-7-*O*-glucoside. Their time profiles are depicted in Figure S1 in the Supplementary Materials.

The flavanol quercetin-3-*O*-rutinoside (*m*/*z* 609.1427 [M-H]^−^) could be identified with a reference standard. The time profile shows a rapid decrease in the first two hours of colon incubation. Quercetin-3-*O*-rhamnoside (*m*/*z* 447.0941 [M-H]^−^) and quercetin aglycone (*m*/*z* 301.0341 [M-H]^−^) were also identified with a reference standard. The time profile of quercetin-3-*O*-rhamnoside exhibited a gradual decrease during the colon phase. The compound was not detected after 12 h of colon incubation. Quercetin aglycone was formed in the first two hours of the colon phase. The signal went down slowly during the 72 h of the colon experiment without disappearing completely. In Table 2, all of the identified compounds are summarised by showing the proposed name, molecular formula, experimental and calculated *m*/*z* of the [M-H]^−^ adduct, error, fragments, confidence level of identification, and used reference.

##### Biotransformation of Phenolic Acids

Lastly, some phenolic acids were identified. Ferulic acid (*m*/*z* 193.0495 [M-H]^−^) is formed in the first 2 h of colon incubation. The signal returned to baseline after 42 to 48 h of the colon phase. (iso)Ferulic acid glucoside (*m*/*z* 355.1019 [M-H]^−^) was tentatively identified by the detection of the fragment *m*/*z* 193, which corresponds to ferulic acid after the neutral loss of a glucose moiety (162 Da). This fragmentation pattern was confirmed by HMDB [40]. The signal in the test samples disappeared quickly after 2 h of colon incubation.

**Figure 5:**
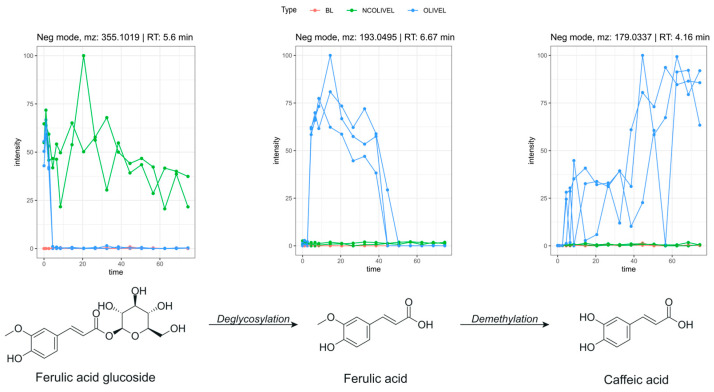
Proposed biotransformation pathway of ferulic acid glucoside with corresponding selected time profiles. Time on the x-axis is expressed in hours. Test samples with olive leaf extract (OLIVEL), negative control samples (NCOLIVEL), and blank samples (BL) are depicted in blue, green, and red, respectively. The metabolites were detected in samples of both age groups.

The identification of caffeic acid (*m*/*z* 193.0495 [M-H]^−^) was confirmed with a reference standard. The time profile showed that caffeic acid was not present at the beginning of the experiment. The signal in the test samples started rising from the start of the colon phase and kept increasing throughout the remainder of the experiment. A proposed biotransformation pathway of ferulic acid glucoside, ferulic acid, and caffeic acid is displayed in Figure 5 together with their time profiles.

Coumaric acid (*m*/*z* 163.039 [M-H]^−^) was tentatively identified. The fragmentation pattern was checked with the spectral database HMDB [40]. The time profile of coumaric acid was similar to the profile of ferulic acid.

Finally, 3-(4-hydroxyphenyl)propionic acid (*m*/*z* 162.0552 [M-H]^−^) and phenylacetic acid (*m*/*z* 135.0446 [M-H]^−^) were detected. 3-(4-hydroxyphenyl)propionic acid could be identified with a reference standard and phenylacetic acid by comparison with the literature [45].

**Table 2:** Summary of identified metabolites of olive leaf extract after in vitro biotransformation by UPLC-ESI-QTOF MS, including retention time, molecular formula, experimental, and calculated *m*/*z* of the [M-H]^−^ adduct, error, fragments, confidence level (CL), and used references. The same metabolites were detected in samples of both age groups.

	Compound	Rt (min)	Molecular Formula	*m*/*z* Experimental	*m*/*z* Calculated	Error (ppm)	Fragments	CL	References
***Olive-specific compounds***									
1	Hydroxytyrosol	2.47	C8H10O3	153.0547	153.0552	−3.27		I	
2	Hydrated product of loganin	3.11	C17H28O11	407.1538	407.1553	−3.68		IV	[41]
3	Oleoside-11-methyl ester	5.43	C17H24O11	403.1238	403.1240	−0.50	223.0602; 179.0550	II	[39,40]
4	Aldehydic decarboxyl elenolic acid	6.23	C10H16O5	215.0915	215.092	−2.32		IV	[39]
5	Hydroxytyrosol acetate	6.46; 7.54; 8.81	C10H12O4	195.0653	195.0657	−2.05		IV	[39,41]
6	Elenolic acid	6.75	C11H14O6	241.0708	241.0712	−1.66		III	
7	Desoxy-elenolic acid	6.77	C11H14O5	225.0757	225.0763	−2.67		IV	[41]
8	Hydroxytyrosol-rutinoside	6.82	C20H30O12	461.1651	461.1659	−1.73		IV	[41]
9	Elenolic acid dialdehyde	7.11	C11H16O6	243.0862	243.0869	−2.88		III	
10	Hydroxy-verbascoside	7.12	C29H36O16	639.1887	639.1925	−5.95		IV	[41]
11	Hydroxylated demethyl derivative of oleuropein	7.22	C23H32O15	547.165	547.1663	−2.38		III	
12	Oleoside/secologanoside	7.24; 8.29	C16H22O11	389.1073	389.1084	−2.83		IV	[41]
13	Demethyloleuropein	7.25	C24H30O13	525.1599	525.1608	−1.71		IV	[41]
14	Verbascoside	7.91	C29H36O15	623.1966	623.1976	−1.60	461.1696; 315.1097	II	[39,42,44,46,47,48,49,50]
15	Isoverbascoside	8.28	C29H36O15	623.1966	623.1976	−1.60	461.1696; 315.1097	II	[39,44]
16	Hydroxytyrosol derivative	8.45	C17H24O6	323.1487	323.1495	−2.48		IV	[41]
17	Oleuropein–glucoside	8.53	C31H42O18	701.2283	701.2293	−1.43	539.1786; 377.1245	II	[42]
18	Hydroxylated derivative of oleuropein	8.81	C25H30O14	553.1557	553.1557	0.00		III	
19	Lactone ester with hydroxytyrosol	8.96	C17H22O6	321.1332	321.1338	−1.87	185.0821	II	[39]
20	Elenolic acid dialdehyde epimer linked to hydroxytyrosol-glucoside	9.15	C25H34O13	541.1919	541.1921	−0.37		IV	[41]
21	Hydroxy-methyl-oleuropein	9.26	C26H34O14	569.1874	569.187	0.70		IV	[41]
22	Oleuropein	9.41	C25H32O13	539.1766	539.1765	0.19	403.1246; 377.1243; 307.0823; 275.0909; 223.0611	I	[39,40]
23	Ligstroside	10.24	C25H32O12	523.182	523.1816	0.76	361.1291; 291.0876; 259.0971	II	[39,42]
24	Elenolic acid derivative	9.49; 10.25	C26H36O13	555.2071	555.2078	−1.26		IV	[41]
25	Dimethyl-hydroxy-ocenoyloxy-secologanoside	10.01; 10.55	C26H38O13	557.2223	557.2234	−1.97		IV	[41]
26	Oleuropein aglycone	10.47	C19H22O8	377.1229	377.1236	−1.86	241.0707; 195.0650	II	[39,40]
27	Derivative of oleuropein aglycone	10.67	C19H24O8	379.1383	379.1393	−2.64	243.0883	III	
Flavonoids									
28	Luteolin-7,4-*O*-diglucoside	6.31	C27H30O16	609.1445	609.1456	−1.81	285.0410; 447.0996	II	[42,50]
29	Apigenin-rhamnosyl-acetyl- glucoside	6.51; 7.90; 8.28	C29H32O15	619.1634	619.1663	−4.68		IV	[41]
30	Quercetin-3-*O*-rutoside	7.29	C27H30O16	609.1427	609.1456	−4.76		I	
31	Luteolin-7-*O*-rutinoside	7.53	C27H30O15	593.15	593.1507	−1.18	285.0407	II	[42,48,50]
32	Luteolin-hexosyl-rhamnoside	7.79; 8.28	C27H30O15	593.1493	593.1507	−2.36		IV	[41]
33	Luteolin-7-*O*-glucoside	7.65	C21H20O11	447.0927	447.0927	0.00	285.0398	I	
34	Apigenin-7-*O*-rutinoside	8.25	C27H30O14	577.1559	577.1557	0.35	269.0450	II	[40,42,48,50]
35	Quercetin-3-*O*-rhamnoside	8.43	C21H20O11	447.0941	447.0927	3.13		I	
36	Apigenin-7-*O*-glucoside	8.46	C21H20O10	431.0982	431.0978	0.93	269.045	I	
37	Chrysoeriol-7-*O*-glucoside	8.74	C22H22O11	461.1098	461.1083	3.25	446.0844; 283.0240; 255.0296	II	[42,44]
38	Luteolin	10	C15H10O6	285.0394	285.0399	−1.75		I	
39	Quercetin	10	C15H10O7	301.0341	301.0348	−2.33		I	
40	Apigenin	10.79	C15H10O5	269.0449	269.045	−0.37		I	
Phenolic acids									
41	Phenyl acetic acid	4.15	C8H8O2	135.0446	135.0446	0.00		II	[45]
42	Caffeic acid	4.16	C9H8O4	179.0342	179.0344	−1.12		I	
43	(iso)Ferulic acid-glucoside	5.6	C16H20O9	355.1019	355.1029	−2.82	193.0482	II	[40]
44	3-(4-hydroxyphenyl)propionic acid	5.64	C9H10O3	165.0547	165.0552	−3.03		I	
45	Coumaric acid	5.78	C9H8O3	163.0390	163.0395	−3.07	119.0491	III	[40]
46	Ferulic acid	6.67	C10H10O4	193.0495	193.0501	−3.11		I	

#### 3.2.3. Controls

While the viable cell count after 72 h of colon incubation averaged around 10^8^–10^9^ CFU/mL in all sample types in both experiments, the viable CFU/mL at the start of the colon phase of the experiment was significantly higher in the elderly experiment compared to the young experiment (*p*-value 0.002, Mann–Whitney U test). Only a significant increase was observed between the start and end of the colon phase in the olive leaf extract samples of the experiment with the healthy young population (*p*-value 0.026, independent samples T test).

The results of the PC samples showed that chlorogenic acid was broken down after 12 to 18 h of colon incubation in both age groups. In general, it can be observed that biotransformation occurred more slowly during the experiment with the young population. Although all expected metabolites could be detected, there was a distinct difference in the formation of 3-(4-hydroxyphenyl)propionic acid between the two age groups. The conversion of its precursor, 3,4-dihydroxyphenylpropionic acid, happened only sparingly in the experiment on the young. Time profiles of the biotransformation of chlorogenic acid and its metabolites can be found in the Supplementary Materials, Figure S2.

### 3.3. Comparison Between Age Groups

#### 3.3.1. Comparison of Metabolite Profiles

A comparison of the identified metabolites between the two age groups was made based on the difference in the peak area of each time point, the difference in the area under the curve (AUC) of the time profile, and the visual inspection of the general course of the time profile.

**Figure 6:**
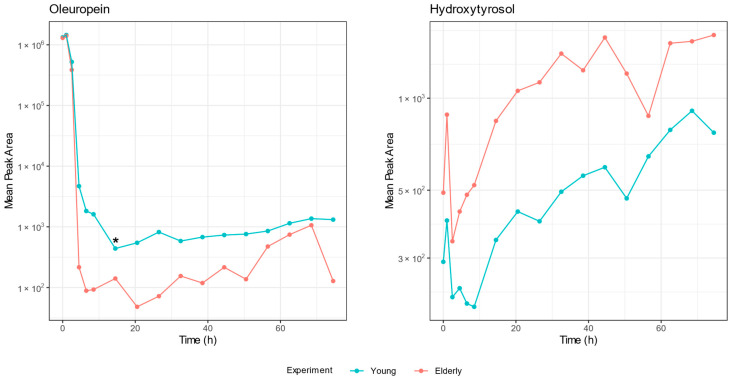
Time profiles of oleuropein and hydroxytyrosol constructed from the peak areas of the compounds detected in samples of the oleuropein biotransformation experiment. Logarithmic transformation of the peak area is plotted on the y-axis and time on the x-axis. The time profiles of the different age groups are depicted in blue (young) and red (elderly). The mean detected peak area of oleuropein after 12 h of colon incubation was significantly different between the experiments and is marked with an asterisk (*) (*q*-value = 0.032).

**Figure 7:**
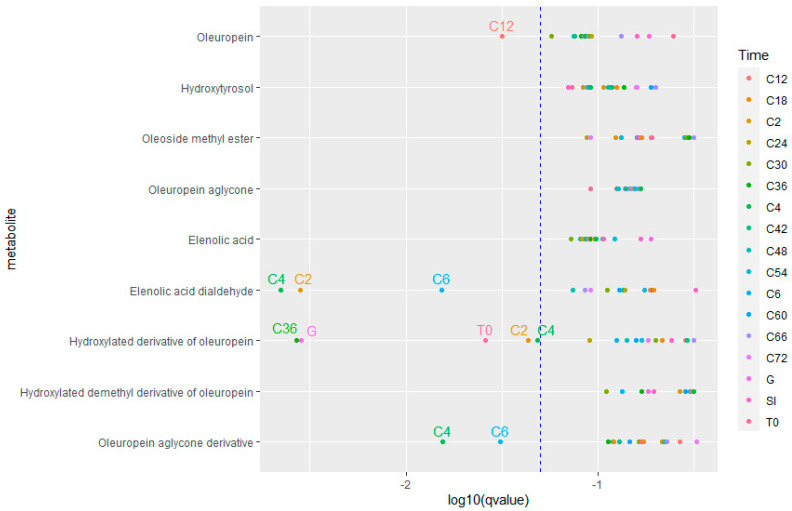
Dot plot of the q values of the comparison of the metabolite peak area between two age groups for each time point of the oleuropein biotransformation experiment. The *q*-value is plotted on the x-axis on a logarithmic scale. Dots on the left side of the blue dotted line have a *q*-value < 0.05.

**Figure 8:**
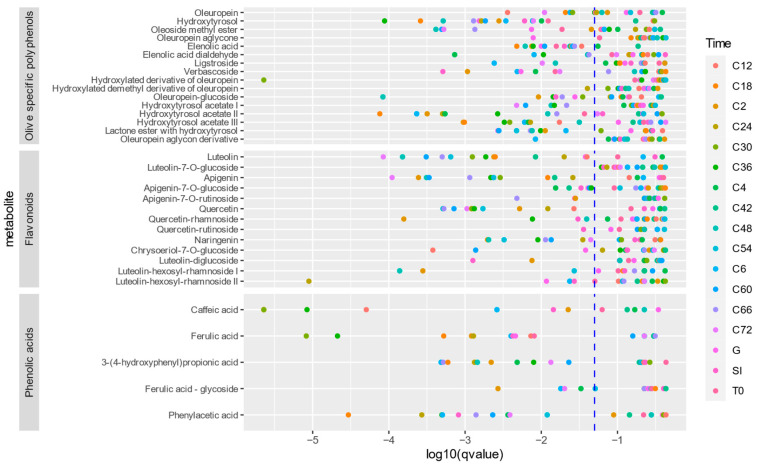
Dot plot of the q values of the comparison of the metabolite peak area between two age groups for each time point of the olive leaf extract biotransformation experiment. The *q*-value is plotted on the x-axis on a logarithmic scale. Dots on the left side of the blue dotted line have a *q*-value < 0.05.

A first observation was that the same metabolites were identified in the samples of both age groups, indicating that there was no distinction in the formation of different metabolites between the young and elderly age groups. To compare the amount of metabolite formation, a time profile was constructed using the peak area. Examples of the time profiles of oleuropein and hydroxytyrosol can be found in Figure 6. Figure 7 shows a dot plot of the *q*-values (*p*-value after correction for multiple testing) resulting from the comparison of the oleuropein biotransformation experiment. Only a few time points were significantly different, and if they were, it was mainly the early colon time points that had a *q*-value lower than 0.05. Figure 8 displays the outcome of the comparison in the experiment with olive leaf extract. In this case, many more time points were significant, but no consistent pattern was observed regarding significant compounds or time points.

To note the differences in the time profiles as a whole, the AUC of the time profiles of the two age groups was compared. The biotransformation of oleuropein gave only a significant result of the time profile of oleuropein itself (*p* < 0.001). The olive leaf extract biotransformation had significantly different AUCs in the cases of flavonoid aglycones, such as apigenin (*p* < 0.001), luteolin (*p* < 0.001), naringenin (*p* < 0.001), and quercetin (*p* = 0.001), and all of the identified phenolic acids, including caffeic acid (*p* < 0.001), ferulic acid (*p* < 0.001), phenylacetic acid (*p* = 0.003), and 3-(4-hydroxyphenyl)propionic acid (*p* = 0.01). The olive-specific polyphenols oleuropein glucoside (*p* = 0.002), oleoside-11-methyl ester (*p* = 0.01), the hydroxylated demethyl derivative of oleuropein (*p* = 0.02), and elenolic acid (*p* = 0.02) had a statistically significant result.

When visually inspecting the time profiles, the same pattern can be observed. As noticeable in Figure 6, there is less breakdown and less formation in the samples of the young population.

#### 3.3.2. Comparison of Bacterial Composition

**Figure 9:**
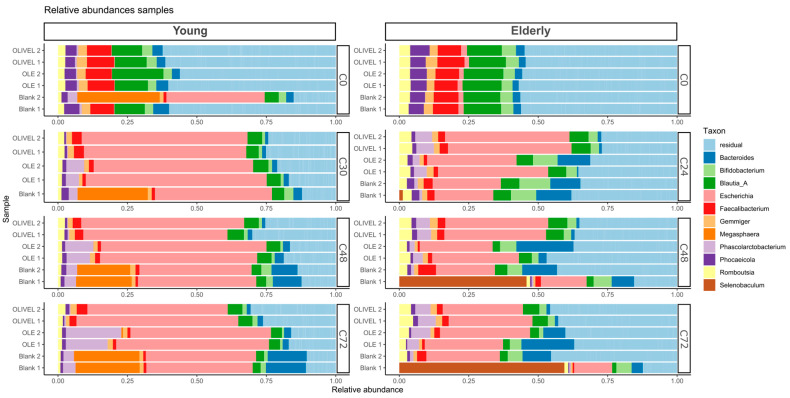
Bar plot of relative abundances of samples taken at the start of the colon phase (C0) and after 24 h (C24) or 30 h (C30), 48 h (C48), and 72 h (C72) of colon incubation for the young (**left**) and elderly (**right**) biotransformation experiment. The eleven most abundant genera are depicted separately.

After sequencing the C0, C30, C48, and C72 samples of the young age group and the C0, C24, C48, and C72 samples of the elderly age group, quality control of the data was executed. The data contained a very low amount of non-bacterial DNA; these were removed from the data set. Samples with less than 1000 reads were omitted. This was the case for the second replicate of the blank sample taken after 30 h of colon incubation during the experiment of the young age group. After the elimination of this sample, the average amount of reads per sample was 24,771 with a minimum of 6152 and a total of 1164,280 reads. A stacked barplot of the relative abundances in the sample was plotted and is depicted in Figure 9. The eleven most abundant genera are displayed separately, and the remaining genera are depicted as ‘other’. During the experiment of the young age group, *Escherichia* dominated all samples at the expense of all other genera after 30 h of colon incubation. Apart from a slight increase in the abundance of *Bacteroides*, *Megasphaera*, and *Phascolarctobacterium* in the blank samples, the situation remained stable. Noteworthy is the presence of *Megasphaera* solely in the blank samples. A less extensive change between the start and the other time points can be observed in the experiment of the elderly age group. Comparable to the young age group, the abundance of *Escherichia*, *Bacteroides*, and *Phascolarctobacterium* increases after 24 h. Remarkable is the presence of the genus *Selenobaculum* in one of the two blank samples of the elderly age group. This genus is very abundant after 48 h of colon incubation.

**Figure 10:**
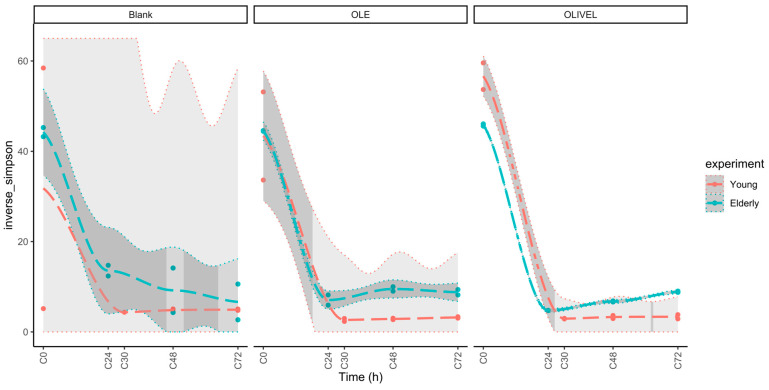
Evolution of inverse-Simpson α-diversity during colon incubation for blank, oleuropein (OLE), and olive leaf extract (OLIVEL) samples for the young (red) and elderly (green) biotransformation experiment. Average values are represented with a long dashed line; 95% confidence intervals are depicted with a dotted line.

The alpha (α) diversity is plotted out in Figure 10. In this plot, a steep drop can be observed after 24 h or 30 h of the colon phase in the experiments of both age groups, independent of the sample type. The α-diversity remained low up until the end of the experiment. In the comparison of the blank samples, a large difference in α-diversity was observed between the duplicates of the young population at C0. Although the course is very comparable between the two age groups, after 24 h or 30 h of colon incubation, the α-diversity in samples of the elderly experiment remained higher.

**Figure 11:**
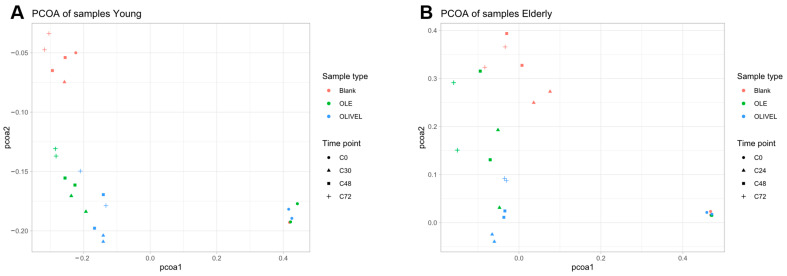
PCoA plots of the Bray–Curtis dissimilarity of the individual oleuropein (OLE), olive leaf extract (OLIVEL), and blank samples, representing the diversity between the different samples taken at the start of the colon phase (C0 ●) and after 24 h (C24 ▲) or 30 h (C30 ▲), 48 h (C48 ■), and 72 h (C72 +) of colon incubation for the young (**A**) and elderly (**B**) biotransformation experiment.

The diversity between the different samples within one experiment (β-diversity) is displayed in the PCoA plots in Figure 11. Figure 11A shows the PCoA plot of the samples of the experiment of the young age group. The samples taken at the start of the colon phase (C0) cluster together, except for one of the blank replicates. Samples of the other time points are not clearly present in separate clusters, regardless of the sample time point, although a certain trend of grouping per sample type can be observed.

The difference between C0 and the other samples in the experiment of the elderly age group (Figure 11B) is comparable to the experiment of the young age group. The C0 samples are clustered, while the other samples are scattered, more than in the young age group. The samples containing olive leaf extract scored higher on the PCoA2 axis, which follows the results of the young age group. However, there is a rather large difference on the PCoA2 axis between the duplicates of the OLE samples of C24, C48, and C72.

**Figure 12:**
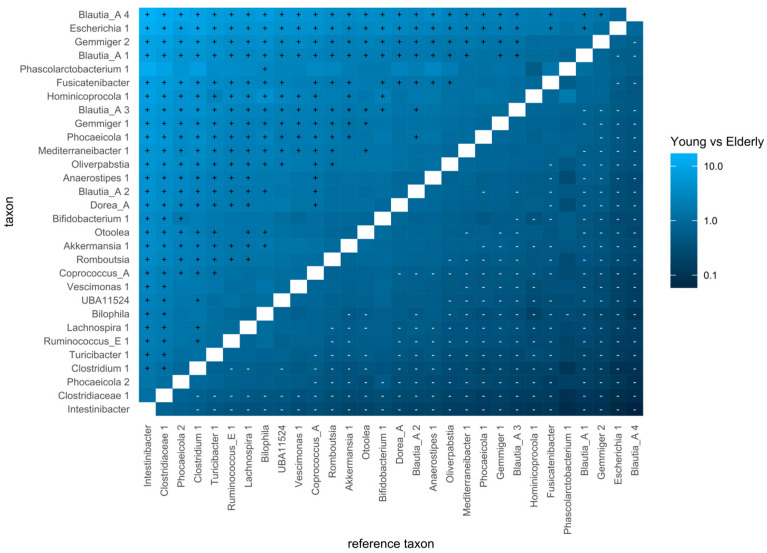
Heatmap of codifferential abundance analysis.

The results of the differential abundance analysis are depicted in a heatmap (Figure 12). It visualises the differential abundance of taxa between the two age groups compared to all other taxa as references. *Blautia_A*, *Escherichia*, *Gimmer*, and *Fusicatenibacter* species were found to be more abundant in the samples of the young age group.

## 4. Discussion

The biotransformation of oleuropein and an olive leaf extract was investigated using an in vitro gastrointestinal simulation model adapted to the physiological conditions of healthy young (20–30 years old) and healthy elderly (≥65 years old) people. A data analysis was performed with an automated workflow, followed by metabolomics profiling using targeted and non-targeted approaches.

The current research findings about the changes in gastrointestinal conditions in the elderly are limited. The more recent reviews on the subject use rather dated references, since most studies date back a few decades. Additionally, the used gastro-intestinal model mimics the gastro-intestinal environment in a fasted state. Therefore, implementing suitable adaptations is more challenging, since many studies focus on the differences between age groups in the fed state or after stimulation.

The literature shows that, in the fed state, the gastric pH is higher, and the gastric emptying is slower in the elderly. There is no clinically relevant difference in basal gastric acid production [26,51,52] and gastric motility [53] in a fasted state between young and older individuals. Concerning pepsin secretion, a study by Feldman et al. showed a reduction in basal pepsin output of 35% in people 65 years or older compared to young individuals (18–34 years old) [26]. Therefore, it was decided to reduce the pepsin concentration by 35%, as observed by Feldman et al., and to keep the duration and pH level in the gastric phase the same in the two experiments. The literature on the ageing pancreas gives controversial results. Conflicting outcomes were obtained when studying the differences in lipase, amylase and protease activity, as well as the total pancreatic secretion. Some studies showed no differences in these parameters between healthy young and healthy older individuals [54,55], while other studies showed a 40% decrease in enzyme output in the elderly after secretin stimulation [56]. Very little research is available on the change in bile acid concentration upon ageing. One study showed a decrease of 38% in bile acid synthesis between people under 40 years of age and individuals over 60 years old [57]. Another study found a 33% decrease in postprandial serum bile acids in older individuals [58]. Given this lack of consensus, the fact that this subject is scarcely studied, that the available literature is dated, and the rather modest effect of the small intestine enzymes on the studied polyphenols [59,60], no alterations in the pancreatin and bile concentrations were made. Also, no overall difference in gut transit time was found between the young and elderly [61,62].

A few months after the execution of the experiment, the COST action INFOGEST published recommendations for static in vitro digestion models adapted to the general older adult population [63]. One of the main objectives of the INFOGEST COST action was to harmonise the protocols of the different static gastro-intestinal simulation models and to provide recommendations for the different parameters, such as pH, duration, and enzyme concentration when simulating oral, gastric, and small intestinal digestion. Aside from the fact that this consensus protocol too describes a simulation model in a fed state, there are two major differences between the recommendations and the used protocol, all situated in the small intestinal phase. First, the consensus protocol favours a decrease of 20% in pancreatic enzymes in the elderly model, despite conflicting results in the literature, with reported differences ranging from 0 to 35%. The second major difference concerns the concentration of bile acids. As discussed, a very scarce amount of the literature is available on this topic. Only two studies published more than 20 years ago [57,58] were considered relevant to substantiate the recommendation to decrease the bile concentration by 33% in the elderly model. The study by Salemans et al. reported a decrease in postprandial serum bile acids, which is less relevant due to the fasted characteristics of the used gastrointestinal simulation model. The results of this study show a limited influence of the small intestinal phase on the studied polyphenols, and the literature suggests that modifications to the chemical structure of the polyphenols are mainly due to the mild alkaline environment in the small intestine, which is not altered in healthy elderly individuals, and cannot be ascribed to interactions with pancreatic enzymes [64]. Taking this into account, it can be concluded that differences between the used protocol and the suggested consensus protocol are of minor relevance.

In the olive leaf extract, phenolic acids, flavonoid glycosides, and some olive-specific polyphenols such as hydroxytyrosol, oleuropein, verbascoside, and ligstroside were identified. The presence of these compounds in olive leaves was previously described in the literature [39,42,46,65,66,67].

The main polyphenol in olive leaves is oleuropein. In the OLE gastrointestinal biotransformation experiment, its time profile suggests that stomach and small intestine conditions do not affect oleuropein. However, a steep drop in signal intensity in the first 2–6 h of the colon phase indicates rapid biotransformation into metabolites by gut microbes. Since a fixed amount of oleuropein was added at the start of the experiment, the rise in signal between T0 and the time point after gastric incubation cannot be ascribed to an increase in concentration but is likely due to the matrix effects that affect the signal intensity during sample analysis. This phenomenon is observed in all of the time profiles of the compounds that were present at the start of the experiment. The time profile of ion *m*/*z* 377.1229 ([M-H]^−^) was tentatively identified as oleuropein aglycon, formed by the loss of the glucose moiety. Its absolute intensity was remarkably lower than oleuropein, implying that this molecule could be very unstable in the sample solution. Its time profile in the OLE and OLIVEL samples followed the same course as the NC samples, meaning that further breakdown is not exclusively facilitated by bacteria. Nevertheless, the signal is notably different from the BL samples during the entirety of the experiment, indicating that the aglycon is continuously being formed and hydrolysed, since the signal never disappeared. The intermediate time profile of oleoside-11-methyl ester reaches a maximum intensity after small intestinal incubation. Next, the gut microbiome breaks it down in the first 12 to 18 h of colon incubation. Oleoside-11-methyl ester is possibly formed by the hydrolytic loss of the hydroxytyrosol moiety of oleuropein. Both hydroxytyrosol and elenolic acid have increasing time profiles, meaning that they are not further metabolised by the gut microbiome. However, a slight decrease in intensity is noticeable for elenolic acid in the test samples after 42 h of colon incubation, and the compound is also detected in the negative control samples, indicating that the formation of elenolic acid cannot entirely be ascribed to the gut microbes. Hydroxytyrosol is not detected in the negative control samples, verifying the essential role of colon bacteria in its formation. Free and conjugated forms can be detected in human plasma after the consumption of olive products, supporting the proposition that hydroxytyrosol might be the final metabolite of the oleuropein metabolic pathway in the colon [47,68,69]. The drops in intensity, visible in the hydroxytyrosol time profile, are likely due to peak detection issues in the automated workflow. The presence of hydroxytyrosol in the samples of these time points was confirmed manually.

Flavanoid glycosides are also present in olive leaf extract. Their time profiles showed a rapid degradation by the colon bacteria within two hours of incubation, forming the corresponding aglycone. The aglycone was then further metabolised by a C-ring cleavage into small phenolic molecules like phenylpropionic acid or phenylacetic acid derivatives and phloroglucinol [17,70]. Although the latter was not detected in the current study, the literature suggests that the cleavage of the C-ring by the gut bacteria results in the formation of phloroglucinol or resorcinol, depending on the substitution of the flavonoid A-ring, leaving the B-ring with a C_2_ or C_3_ fragment to form phenylacetic or phenylpropionic acids [17,71,72]. This was observed in the case of quercetin-3-*O*-rutinoside, apigenin-7-*O*-glucoside, and luteolin-7-*O*-glucoside. *Bacteroides*, *Bifidobacterium*, *Enterococcus*, *Eubacterium*, *Escherichia*, and *Lactobacillus* species have demonstrated the capability to perform *O*-deglycosylation reactions with the mentioned flavonoid glycosides as substrates [73,74,75,76], followed by cleavage of the C-ring facilitated by some *Eggerthella*, *Eubacterium*, and *Flavonifractor* species, yielding phenylacetic or phenylpropionic acids [73,77,78,79].

Hydroxycinnamic acids, like caffeic acid, coumaric acids, and ferulic acids were identified as biotransformation products of olive leaf polyphenols. The ferulic acid glucoside is present in the extract itself and quickly undergoes a deglycosylation reaction by the colon bacteria. The resulting ferulic acid is subsequently converted into caffeic acid through a demethylation reaction.

While the same metabolites are found in the samples of both age groups, a pattern can be observed when comparing the peak areas of the identified compounds between the age groups at each time point. As shown in Figure 6, there is less breakdown and less formation of the metabolites in the young age group. Taking into account the lower viable cell count during the experiment with the faecal pool of the young population, this observation can be readily explained. When comparing the AUCs of the time profiles between the age groups, the compounds that are situated mainly at the later stages of the biotransformation pathway were significantly different. This observation can result from the difference in viable cells between the experiments. Biotransformation products are being formed in a greater quantity in samples with more viable bacteria, with an increasing difference between the two age groups as the experiment progresses, leading to more statistically different profiles of compounds that are metabolised by the gut bacteria. However, since there were significantly different time points for all classes of detected compounds, the observed effect was general and not due to a certain strain that became more dominant during colon incubation.

The investigation of the biotransformation pathway of the PC, chlorogenic acid, shows a slower conversion during the experiment of the younger age group, as depicted in Figure S2 of the Supplementary Materials. A prominent difference can be observed in the dehydroxylation of 3,4-(dihydroxyphenyl)propionic acid to 3-(4-hydroxyphenyl)propionic acid, as this reaction occurred almost exclusively in the elderly age group. Again, this phenomenon can be explained by the difference in viable cells.

The gut microbiota is predominately composed of six phyla, namely *Actinobacteria*, *Bacteroidetes*, *Firmicutes*, *Fusobacteria*, *Proteobacteria*, and *Verrucomicrobia*, among which *Bacteroidetes* and *Firmicutes* are the most abundant [80]. This corresponds with the findings of the 16S rRNA sequencing of the individual faecal samples (Figure 1). The genera *Bacteroides*, *Phocaeicola*, and *Prevotella*, belonging to the *Bacteroidetes* phylum, are the most common. Additionally, *Faecalibacterium* (*Firmicutes*) and *Bifidobacterium* (*Actinobacteria*) are one of the most abundant genera. During ageing, the gut microbiome undergoes some changes. General age-related changes are characterised by a loss of the dominant commensal taxa (e.g., *Prevotella*, *Faecalibacterium*, *Lachnospira,* and *Bifidobacterium*) and an increase in putatively beneficial species like *Akkermansia* and *Butyricicoccus* and pathobionts (e.g., *Eggerthella*, *Streptococcus*, and *Enterobacteriaceae*) [81]. The noticeable decrease in the abundance of *Bifidobacteria* between the samples of young and elderly individuals is consistent with the findings in the literature [81]. The results suggest that *Bacteroides* are more common in younger individuals, while studies report an overall increase in the abundance of the phylum *Bacteroidetes* with ageing [82].

Looking at the evolution of the bacterial composition during the two experiments, the most striking observation is the difference between the start of the experiment (C0) and the other time points. This is reflected in the drop in α-diversity, the clustering of C0 samples in the β-diversity PCoA plots, and the difference in the relative abundance barplots. The considerable increase in abundance of *Escherichia*, likely due to the experimental setup being favourable for this species after 24 h or 30 h of colon incubation explains the sudden drop in α-diversity and also the clustering of the C0 samples and their distance to the other samples in the PCoA plots (Figure 11A). The large distance between the duplicates of the C0 sample on the PCoA plot of the young experiment is due to the majorly different relative abundance. The abundance profile of one of the blank duplicates resembles that of the blank samples of later time points. This can be explained by cross-contamination or an experimental error during sequencing. This also clarifies the difference in α-diversity between the two duplicates in the young age group.

When looking at the relative abundance plots (Figure 9), a distinction in relative abundance in *Bacteroides* and *Bifidobacterium* in the C24, C48, and C72 OLE samples of the elderly age group can be the reason for the different PCoA2 scoring of the duplicates.

Megasphaera is only present in the BL samples of the young population, suggesting that oleuropein and/or other constituents of the olive extract result in less advantageous experimental conditions for this species. The presence of *Selenobaculum* in one of the BL duplicates of the elderly experiment explains the larger distance between the duplicates on the PCoA plot (Figure 11B).

The relative abundance profile underwent significant changes in the first 24 h of colon incubation in this gastrointestinal simulation model, making this period the most relevant and representative of the in vivo situation.

The codifferential analysis shows a larger abundance of *Escherichia* in the samples of the young population, which is to be expected given its explosive growth in the C30, C48, and C72 samples. The genera *Blautia-a* and *Gemmiger* seem to be more abundant in samples of the young population. They belong to the families *Lachnospiraceae* and *Ruminococcaceae,* respectively. Their decreasing abundance along with ageing was previously described in the literature [83].

Remarkable is the presence of the genus *Selenobaculum* in one of the blank samples of the elderly pool. Very little information is available about this genus. Searches on the Genome Taxonomy Database (GTDB) [33] revealed that this genus, previously CABIZH01, is renamed to *Selenobaculum*. The species *Selenobaculum gbiensis* sp. nov. was first isolated from a faecal sample of a 26-year-old patient with Crohn’s disease [84]. Since the same pool of the faecal suspension was used in all BL, NC, OLE, and OLIVEL samples during the experiment, the enormous abundance in only one sample was unexpected. Further research is necessary to assess the impact of this genus on the gut microbiome.

## 5. Conclusions

The current study investigated the biotransformation of oleuropein and an olive leaf extract using an in vitro gastrointestinal simulation model with a colon phase adapted to two age groups (20–30 years old and ≥65 years old) of healthy volunteers. The existing literature on the influence of ageing on the gastrointestinal system is limited, highlighting the need for more in-depth research.

The majority of biotransformation reactions occurred within the first 24 h of colon incubation. Investigations into the bacterial composition during the experiment revealed a major shift in relative abundance in the first 24 h, making it the most representative. Mainly deglycosylation, hydrolysis, flavonoid ring cleavage, methylation, and demethylation reactions of the studied metabolites were observed. Samples from the younger age group exhibited less extensive metabolite breakdown and formation, most probably due to a lower viable cell count.

Hydroxytyrosol, the key biotransformation product of oleuropein, was not further metabolised by the gut bacteria, indicating that it is the final metabolite in the colon.

Although many different metabolites were detected, the same metabolites were identified in the samples of both age groups, indicating that the potential for polyphenols to exert their health-promoting benefits persists in healthy older individuals.

## Figures and Tables

**Table 1:** Summary of identified metabolites of oleuropein after in vitro biotransformation by UPLC-ESI-QTOF MS, including retention time, molecular formula, experimental, and calculated *m*/*z* of the [M-H]^−^ adduct, error, fragments, confidence level (CL), and used references. The same metabolites were detected in samples of both age groups.

	Compound	Rt (min)	Molecular Formula	*m*/*z* Experimental	*m*/*z* Calculated	Error (ppm)	Fragments	CL	References
1	Hydroxytyrosol	2.47	C_8_H_10_O_3_	153.0547	153.0551	−2.61		I	
2	Oleoside-11-methyl ester	5.43	C_17_H_24_O_11_	403.1238	403.1240	−0.50	223.0602; 179.0550	II	[39,40]
3	Elenolic acid	6.75	C_11_H_14_O_6_	241.0708	241.0712	−1.66		III	
4	Elenolic acid dialdehyde	7.11	C_11_H_16_O_6_	243.0862	243.0869	−2.88		III	
5	Hydroxylated demethyl derivative of oleuropein	7.22	C_23_H_32_O_15_	547.165	547.1663	−2.38		III	
6	Hydroxylated derivative of oleuropein	8.81	C_25_H_30_O_14_	553.1557	553.1557	0.00	403,1246; 223.0611	III	
7	Oleuropein	9.41	C_25_H_32_O_13_	539.1764	539.1764	0.00	403,1246; 377.1243; 307.0823; 275.0909; 223.0611	I	[39,40]
8	Oleuropein aglycone	10.47	C_19_H_22_O_8_	377.1229	377.1236	−1.86	241.0707; 195.0650	II	[39,40]
9	Derivative of oleuropein aglycone	10.67	C_19_H_24_O_8_	379.1383	379.1393	−2.64	243.0883	III	

## Data Availability

The datasets of the 16S rRNA sequencing have been deposited in the European Nucleotide Archive (ENA) at AMBL-EBI under accession number PRJEB80908 (https://www.ebi.ac.uk/ena/browser/view/PRJEB80908 (accessed on 8 October 2024)). Other data are available upon request.

## Supplementary Materials

The following supporting information can be downloaded at https://www.mdpi.com/article/10.3390/metabo15010026/s1: Section S1: Inclusion Criteria Faecal Donors; Section S2: Positive Control—Chlorogenic Acid; Section S3: Extract Identification; Table S1: Identified Compounds in Olive Leaf Extract; Figure S1: Time Profiles Flavonoids; Figure S2: Metabolic Pathway and Time Profiles Chlorogenic Acid. References [85,86] are cited in Supplementary Materials.
