# Supplementary material for: Comparison of In Vitro Biotransformation of Olive Polyphenols Between Healthy Young and Elderly
Source: Metabolites. 2025 Jan 7;15(1):26. doi: 10.3390/metabo15010026 (PMC11766994; doi:10.3390/metabo15010026)
Supplement: Supplementary file 1 [file metabolites-15-00026-s001.zip › metabolites-3370362-supplementary.pdf]

Comparison of *in vitro* biotransformation of Olive Polyphenols Between Healthy Young and Elderly

Lauwers Stef [1], Weyns Anne-Sophie [1], Breynaert Annelies [1], Van Rillaer Tim [2], Van Huynegem Valerie [1], Fransen Erik [3], Bittremieux Wout [4], Lebeer Sarah [2], Tuenter Emmy [1], Hermans Nina [1]

1) Natural Products & Food Research and Analysis – Pharmaceutical Technology (NatuRAPT), Department of Pharmaceutical Sciences, University of Antwerp, Universiteitsplein 1, 2610 Wilrijk, Antwerp, Belgium

2) Laboratory of Applied Microbiology and Biotechnology, Department of Bioscience Engineering, University of Antwerp, Groenenborgerlaan 171, 2020 Antwerp, Antwerp, Belgium

3) Faculty of Pharmaceutical, Biomedical, and Veterinary Sciences, University of Antwerp, Universiteitsplein 1, 2610 Wilrijk, Antwerp, Belgium

4) ADReM Data Lab, Department of Computer Science, University of Antwerp, Middelheimlaan 1, 2020 Antwerp, Antwerp, Belgium

Supplementary material

Contents

S1 Inclusion Criteria Faecal Donors 2

S2 Positive Control – Chlorogenic Acid 3

S3 Extract Identification 3

Table S1 Identified Compounds in Olive Leaf Extract 5

Figure S1 Time Profiles Favonoids 6

Figure S2: Metabolic Pathway and Time Profiles Chlorogenic Acid 7

References 8

S1 Inclusion Criteria Faecal Donors

- Men or Women aged 20 to 30 years or ≥ 65 years;
- BMI between 18.5 and 25;
- Waist circumference < 88 cm (women), < 102 cm (men);
- Not pregnant or menstruating at time of donation;
- Non-smoker: min. 5 years in advance of donation;
- No vegetarian or vegan;
- No acute condition (e.g. diarrhoea, fever, vomiting) at time of donation;
- No chronic condition requiring medical attention or medication at time of donation;
- No gastro-intestinal illnesses or disorders (e.g. Crohn's disease, Ulcerative colitis, IBS, *Clostridium difficile* colitis, stomach ulcers, *Helicobacter pylori* infection,…) or gastro-intestinal surgical procedures (gastric bypass);
- No history of typhoid fever;
- No allergies;
- No immune-suppressing medication or chemotherapeutics;
- No travel to areas with risk of traveller's diarrhoea in last 6 months prior to donation;
    - Africa, except South Africa,
    - The Middle East,
    - Asia, except Japan and Thailand,
    - Oceania, except Australia and New Zealand,
    - Central America, South America and the Caribbean, except for Argentina and Chile.
- No vaccination in last 3 months prior to donation;
- No illicit drug use;
- No use of pre-, pro-, or antibiotics in last 3 months before donation;
- Normal defecation urge (1-3 times a day).

S2 Positive Control – Chlorogenic Acid

To assess the validity of the experiment, a positive control (PC) sample, containing 75 mg of chlorogenic acid, was used alongside the other samples. Samples of the simulated digestion of the positive control were screened for five metabolites using analytical standards of chlorogenic acid (*m/z* 535.0872 $[M-H]^-$), quinic acid (*m/z* 191.0556 $[M-H]^-$), caffeic acid (*m/z* 179.0344 $[M-H]^-$), 3,4-dihydroxyphenylpropionic acid (*m/z* 181.0501 $[M-H]^-$), and 3-(4-hydroxyphenyl)propionic acid (*m/z* 165.0557 $[M-H]^-$). The formation of these metabolites was used as confirmation of *in vitro* biotransformation.

S3 Extract Identification

With the described method, a total of 16 phenolic compounds were identified tentatively or with a reference standard. Mainly flavonoids, phenolic acids, secoiridoids and other olive-specific polyphenols were characterised. In **Table S1** Identified Compounds in Olive Leaf Extract

Table S1, the identified compounds are summarized showing the proposed name, molecular formula, experimental and calculated *m/z* of the $[M-H]^-$ adduct, error, fragments, confidence level of identification, and used reference.

The olive-specific compounds hydroxytyrosol (*m/z* 153.0550 $[M-H]^-$) and oleuropein (*m/z* 539.176 $[M-H]^-$) were identified with a reference standard. Hydroxytyrosol-glucoside (*m/z* 315.1090 $[M-H]^-$) and oleuropein-glucoside (*m/z* 701.2283 $[M-H]^-$) were tentatively identified by the presence of the corresponding aglycon, respectively hydroxytyrosol (*m/z* 153) and oleuropein (*m/z* 539), each with a difference of 162 Da with the parent ion corresponding with a neutral loss of a glucose moiety. Both compounds were previously described in literature [1,2]. Verbascoside and isoverbascoside were identified by the detection of a signal with *m/z* 623.1966 ($[M-H]^-$) and its fragments *m/z* 461 and *m/z* 315 formed by the loss of a caffeoyl moiety (162 Da) and the loss of a rhamnose moiety (146 Da), respectively (Kundisová et al., 2020; Leouifoudi et al., 2015; Moreno-González et al., 2020; Quirantes-Piné et al., 2013; Talhaoui et al., 2014; MassBank). The distinction between verbascoside and isoverbascoside could be made by the order of elution. A comparable method, developed by [8], demonstrated that verbascoside eluted earlier than isoverbascoside. Ligstroside (*m/z* 523.1819 $[M-H]^-$) could be tentatively identified based on the fragments *m/z* 361, 291 and 259, which were previously reported in literature [2,8]. The fragment ion *m/z* 361 matches with the neutral loss of a sugar moiety (162 Da).

Flavonoids apigenin-7-*O*-glucoside (*m/z* 431.097 $[M-H]^-$), luteolin-7-*O*-glucoside (*m/z* 447.0927 $[M-H]^-$) and quercetin-3-*O*-rutinoside (*m/z* 609.1445 $[M-H]^-$) were identified with a reference standard. The identification of luteolin-7-*O*-rutinoside (*m/z* 593.1503 $[M-H]^-$) was based on the presence of the aglycon fragment ion *m/z* 285 which is resulting from the loss of the rutinoside moiety. [2,4,6]. The fragment ions *m/z* 447 and *m/z* 285 support the identification of luteolin-7,4-*O*-diglucoside (*m/z* 609.1445 $[M-H]^-$). The fragment ions are produced by the neutral loss of a first and second sugar moiety respectively [2,6]. The loss of a rutinoside moiety produces the fragment ion at *m/z* 269, corresponding with apigenin, and therefore verifying the annotation of apigenin-7-*O*-rutinoside to the *m/z* 577.1559 $[M-H]^-$ ion [2,4,6]. The fragment ions were confirmed by searches on the Human Metabolome Database (HMDB; https://hmdb.ca/) [9]. The presence of chrysoeriol-7-*O*-glucoside (*m/z* 461.1098 $[M-H]^-$) could be verified by the production of the *m/z* 299 fragment, formed by the loss of a sugar moiety [2]. The other fragment ions were confirmed by searches on MassBank (https://massbank.eu/MassBank/) [7,10].

The phenolic acid vanillic acid (*m/z* 167.0338 $[M-H]^-$) was identified with a reference standard. (iso)Ferulic acid-glucoside (*m/z* 355.1019 $[M-H]^-$) could be tentatively identified. The neutral loss of a sugar moiety resulted in the fragment ion at *m/z* 193 which corresponds with (iso)ferulic acid [11].

Table S1 Identified Compounds in Olive Leaf Extract

Table S1 *Summary of identified compounds in olive leaf extract by UPLC-ESI-QTOF MS, including retention time, molecular formula, experimental and calculated m/z of the [M-H]- adduct, error, fragments, confidence level (CL), and used references.*

	Compound	Rt (min)	Molecular formula	*m/z* experimental	*m/z* calculated	Error (ppm)	Fragments	CL	Reference
Olive-specific compounds									
**1**	Hydroxytyrosol	2.52	$C_8H_{10}O_2$	153.0550	153.0551	-0.65	123.0439	I	
**2**	Hydroxytyrosol glucoside	2.53	$C_{14}H_{20}O_8$	315.1090	315.1080	3.17	153.0535	II	[1]
**3**	Verbascoside	7.91	$C_{29}H_{36}O_{15}$	623.1966	623.1976	-1.60	461.1696; 315.1097	II	[2–6,8,10,12]
**4**	Isoverbascoside	8.29	$C_{29}H_{36}O_{15}$	623.1966	623.1976	-1.60	461.1696; 315.1097	II	[8,10]
**5**	Oleuropein-glucoside	8.56	$C_{31}H_{42}O_{18}$	701.2283	701.2293	-1.43	539.1786; 377.1245	II	[2]
**6**	Oleuropein	9.42	$C_{25}H_{32}O_{13}$	539.1766	539.1764	0.37	403.1246; 377.1243; 307.0823; 275.0909; 223.0611	I	[8,9]
**7**	Ligstroside	10.25	$C_{25}H_{32}O_{12}$	523.1819	523.1825	-1.15	361.1291; 291.0876; 259.0971	II	[2,8]
Flavonoids									
**8**	Luteolin-7,4-*O*-diglucoside	6.3	$C_{27}H_{30}O_{16}$	609.1445	609.1456	-1.81	285.0410; 447.0996	II	[2,6]
**9**	Quercetin-3-*O*-rutinoside	7.29	$C_{27}H_{30}O_{16}$	609.1427	609.1455	-4.60	301.0336	I	
**10**	Luteolin-7-*O*-rutinoside	7.53	$C_{27}H_{30}O_{15}$	593.1503	593.1501	0.34	285.0407	II	[2,4,6]
**11**	Luteolin-7-*O*-glucoside	7.65	$C_{21}H_{20}O_{11}$	447.0927	447.0927	0.00	285.0398	I	
**12**	Apigenin-7-*O*-rutinoside	8.25	$C_{27}H_{30}O_{14}$	577.1559	577.1557	0.35	269.0450	II	[2,4,6,9]
**13**	Apigenin-7-*O*-glucoside	8.48	$C_{21}H_{20}O_{10}$	431.0982	431.0978	0.93	269.0450	I	
**14**	Chrysoeriol-7-*O*-glucoside	8.76	$C_{22}H_{22}O_{11}$	461.1098	461.1084	3.04	446.0844; 299.0555; 283.0240; 255.0296	II	[2,10]
Phenolic acids									
**15**	Vanillic acid	4.17	$C_8H_8O_4$	167.0338	167.0344	-3.59		I	
**16**	(iso)Ferulic acid-glucoside	5.6	$C_{16}H_{20}O_9$	355.1019	355.1034	-4.22	193.0482	II	[11]

Figure S1 Time Profiles Flavonoids

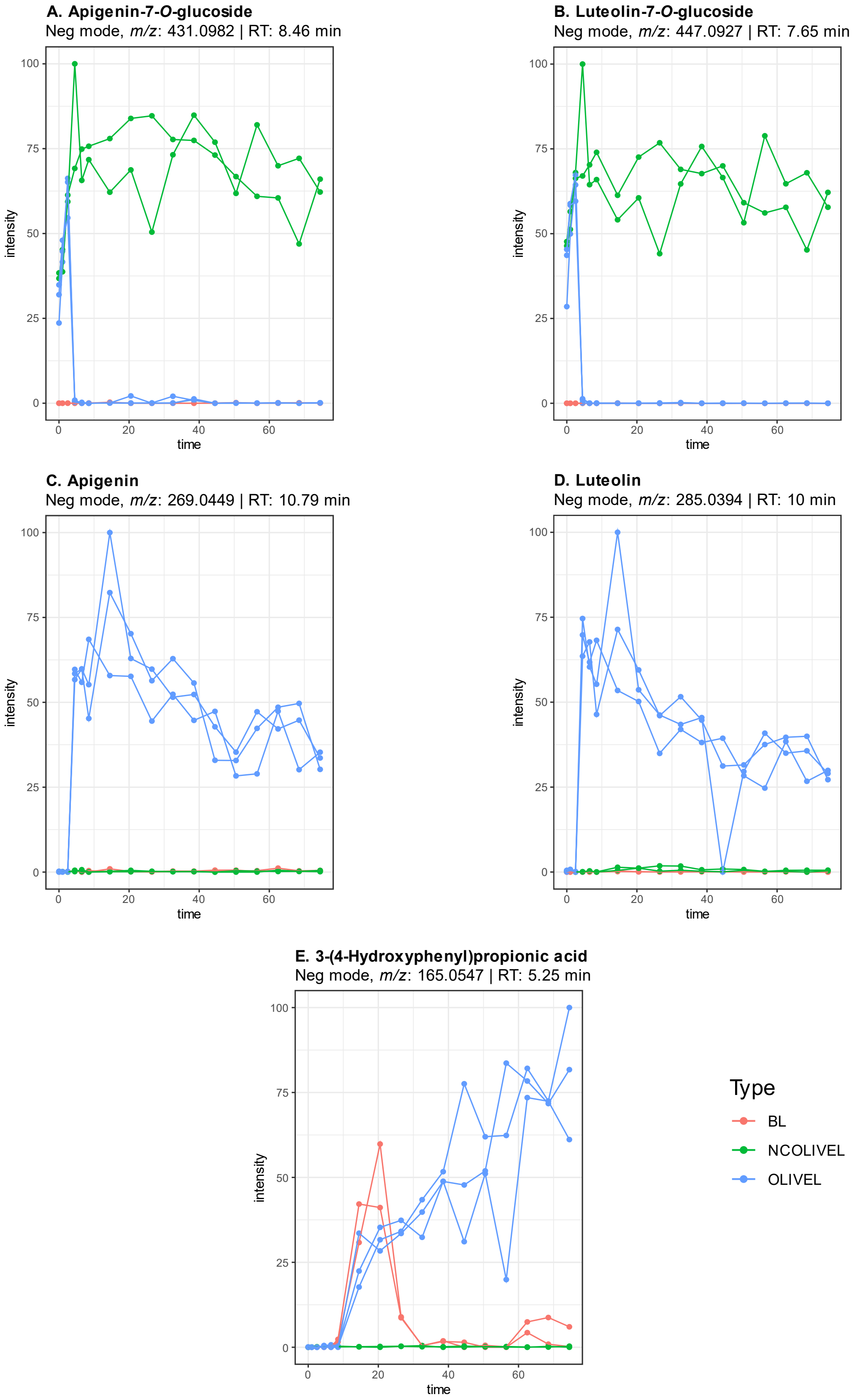

Figure S1 *Time profiles of apigenin-7-O-glucoside (A), luteolin-7-O-glucoside(B), apigenin (C), luteolin (D), and 3-(4-hydroxyphenyl)propionic acid (E). Time on x-axis is expressed in hours and relative intensity of the detection signal is plotted on the y-axis. Test samples with olive leaf extract (OLIVEL), negative control samples (NCOLIVEL), and blank samples (BL) are depicted in blue, green and red respectively.*

Figure S2: Metabolic Pathway and Time Profiles Chlorogenic Acid

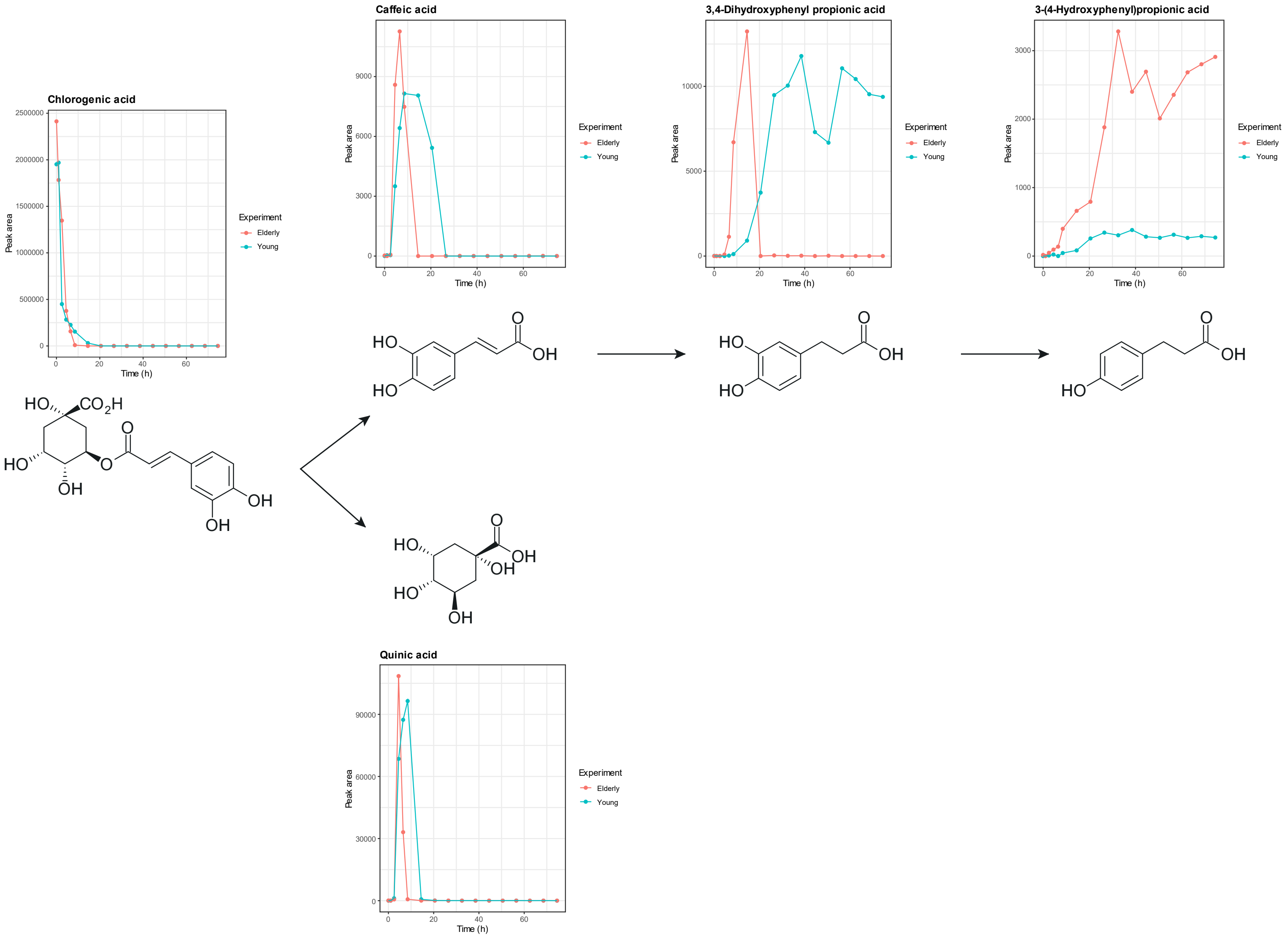

__Figure S2__ Proposed biotransformation pathway of chlorogenic acid with corresponding time profiles used as control of biotransformation. Time on the x-axis is expressed in hours. Time profiles of the elderly and young are depicted in red and green respectively.

References

[1] Ramos P, Santos SAO, Guerra ÂR, Guerreiro O, Felício L, Jerónimo E, et al. Valorization of olive mill residues: Antioxidant and breast cancer antiproliferative activities of hydroxytyrosol-rich extracts derived from olive oil by-products. Ind Crops Prod 2013;46:359–68. https://doi.org/10.1016/J.INDCROP.2013.02.020.

[2] Talhaoui N, Gómez-Caravaca AM, León L, De la Rosa R, Segura-Carretero A, Fernández-Gutiérrez A. Determination of phenolic compounds of 'Sikitita' olive leaves by HPLC-DAD-TOF-MS. Comparison with its parents 'Arbequina' and 'Picual' olive leaves. LWT - Food Science and Technology 2014;58:28–34. https://doi.org/10.1016/J.LWT.2014.03.014.

[3] Kundisová I, Juan ME, Planas JM. Simultaneous Determination of Phenolic Compounds in Plasma by LC-ESI-MS/MS and Their Bioavailability after the Ingestion of Table Olives. J Agric Food Chem 2020;68:10213–22. https://doi.org/10.1021/ACS.JAFC.0C04036/ASSET/IMAGES/LARGE/JF0C04036_0002.JPEG.

[4] Leouifoudi I, Harnafi H, Zyad A. Olive Mill Waste Extracts: Polyphenols Content, Antioxidant, and Antimicrobial Activities. Adv Pharmacol Sci 2015;2015. https://doi.org/10.1155/2015/714138.

[5] Moreno-González R, Juan ME, Planas JM. Table olive polyphenols: A simultaneous determination by liquid chromatography–mass spectrometry. J Chromatogr A 2020;1609:460434. https://doi.org/10.1016/J.CHROMA.2019.460434.

[6] Quirantes-Piné R, Lozano-Sánchez J, Herrero M, Ibáñez E, Segura-Carretero A, Fernández-Gutiérrez A. HPLC–ESI–QTOF–MS as a Powerful Analytical Tool for Characterising Phenolic Compounds in Olive-leaf Extracts. Phytochemical Analysis 2013;24:213–23. https://doi.org/10.1002/PCA.2401.

[7] Horai H, Arita M, Kanaya S, Nihei Y, Ikeda T, Suwa K, et al. MassBank: a public repository for sharing mass spectral data for life sciences. Journal of Mass Spectrometry 2010;45:703–14. https://doi.org/10.1002/JMS.1777.

[8] Kanakis P, Termentzi A, Michel T, Gikas E, Halabalaki M, Skaltsounis AL. From Olive Drupes to Olive Oil. An HPLC-Orbitrap-based Qualitative and Quantitative Exploration of Olive Key Metabolites. Planta Med 2013;79:1576–87. https://doi.org/10.1055/S-0033-1350823.

[9] Wishart DS, Guo AC, Oler E, Wang F, Anjum A, Peters H, et al. HMDB 5.0: the Human Metabolome Database for 2022. Nucleic Acids Res 2022;50:D622. https://doi.org/10.1093/NAR/GKAB1062.

[10] Oberacher H, Sasse M, Antignac JP, Guitton Y, Debrauwer L, Jamin EL, et al. A European proposal for quality control and quality assurance of tandem mass spectral libraries. Environ Sci Eur 2020;32:1–19. https://doi.org/10.1186/S12302-020-00314-9/FIGURES/8.

[11] Hong Y, Wang Z, Barrow CJ, Dunshea FR, Suleria HAR. High-Throughput Screening and Characterization of Phenolic Compounds in Stone Fruits Waste by LC-ESI-QTOF-MS/MS and Their Potential Antioxidant Activities. Antioxidants 2021;10:1–22. https://doi.org/10.3390/ANTIOX10020234.

[12] Bongiorno D, Di Stefano V, Indelicato S, Avellone G, Ceraulo L. Bio-phenols determination in olive oils: Recent mass spectrometry approaches. Mass Spectrom Rev 2023;42:1462–502. https://doi.org/10.1002/MAS.21744.
